# Supplementary material for: Towards a concrete landscape: Assessing the efficiency of land consumption in the Greater Accra Region, Ghana
Source: PLoS One. 2022 Jun 7;17(6):e0269120. doi: 10.1371/journal.pone.0269120 (PMC9173643; doi:10.1371/journal.pone.0269120)
Supplement: Attachment — Submitted filename: PONE review.docx [file pone.0269120.s001.docx]

This paper raised a concern of low land consumption efficiency in Greater Accra Region, by mapping the land cover change in 30 years, the authors calculated the land consumption efficiency using UN habitat’s LCPC and found that the land consumption in the region was highly inefficient. By running random forest model, the authors also identified variables that are associated with land consumption and predicted the future land cover change. The paper has solid sections in background and data, but relative weak in terms of the method and result interpretations.

Overall, the paper may have significant implications for the urban development in Greater Accra Region, especially how to balance the development of urban and population growth. However, I would say that neither the method nor the concluding remarks offer significant contribution to the existing literature, neither in theoretical nor practical domain. For international readers, we would like some take-away from the paper, but I failed to do so. I would suggest put more focus on the interpretation of the associations between land consumption and the influencing factors, and what can we learn from the results. Also, I am not convinced by the prediction results from the random forest model, as the method and model are not well introduced, and the results still require more in-depth interpretation.

Some other questions or comments are as follow:

- Line 174: maybe give some brief introduction to each term, such as what is cultivated and managed terrestrial areas, or just provide a link to the FAO documents.
- Line 180, I don’t quite understand how you decided to use the 1987 and 2017 maps to detect land cover change, as you said the accuracy of 1987 is the weakest.
- Line 184: the efficiency of land is calculated here as the ratio between two growth rate, but why later was defined as population density (land consumption per capita)? Such as 1-150 people per hectare as inefficient. Also, I did not see the results of the LCRPGR in the rest of the paper, but only the LCPC.
- Line 204: although you listed the references that support the selection of independent variables, I suggest add some explanations, especially the theoretical underpinnings behind the potential associations.
- Line 237: should be Table 3.
- Line 247, use cumulative “AS”, and the number should be 106580.8
- Reformate Table 2 and Table 4 to fit the pages.
- Line 278: the random forest modelling should be provided with more details. I am not an expert on random forest model and correct me if I am wrong. I understand that you were using the random forest to regress the correlations between land cover change and the influencing factors, and then using such correlations to predict the future change of land cover under “business as usual” scenario, however, more information should be provided, such as what is the target year of your prediction, have you set any thresholds of the influencing factors, such as the population growth (such as the world population projected by UN), climate change (maybe also from UN’s climate report?).
- Line 283: the interpretation of the Fig 5, while I can tell from the Figure that population has the highest importance, but the text said poverty index has the highest level of importance.
- I have read paper using population projection to predict land use change, or verse vise, but why do you think a random forest that includes all factors in the model is better?
- Line 303: it should be “Leo-Breiman”.
- Line 342: the authors pointed out the planning and protection policies intensified the land consumption, but how to include such influences in the predicting model?
- What is the unit of analysis, especially when calculating the land efficiency? grid cells?
- Actually, you were using the land consumption per capita (LCPC) from UN Habitat (https://unhabitat.org/sites/default/files/2021/08/indicator_11.3.1_training_module_land_use_efficiency.pdf) to calculate the “land use efficiency”, rather than the LCRPGR you mentioned (whether the increase of urbanized land is compatible with the increase of population, line 184). In addition, I did not find the threshold of low, moderately and efficient from the reference you provided (line 188, https://apo.org.au/sites/default/files/resource-files/2018-07/apo-nid182836.pdf).
