# Supplementary material for: Towards a concrete landscape: Assessing the efficiency of land consumption in the Greater Accra Region, Ghana
Source: PLoS One. 2022 Jun 7;17(6):e0269120. doi: 10.1371/journal.pone.0269120 (PMC9173643; doi:10.1371/journal.pone.0269120)
Supplement: Attachment — Submitted filename: Response to Reviewers.docx [file pone.0269120.s002.docx]

**REVIEWER 1**

***Comment 1***

Line 174: maybe give some brief introduction to each term, such as what is cultivated and managed terrestrial areas, or just provide a link to the FAO documents.

***Response 1***

Marked Line 208-226

Unmarked Line 195-210

Brief explanation is provided for each land cover type. Also link to assess in-depth meanings is provided

***Comment 2***

Line 180, I don’t quite understand how you decided to use the 1987 and 2017 maps to detect land cover change, as you said the accuracy of 1987 is the weakest.

***Response 2***

Satellite image for the area starts from 1987. The year 2017 was used because at the time of the study that was the current year and it also provided a 30 year span for the study. The 1987 had few bands compared with the 2005 and 2017 possibly accounting for less accuracy.

***Comment 3***

Line 184: the efficiency of land is calculated here as the ratio between two growth rate, but why later was defined as population density (land consumption per capita)? Such as 1-150 people per hectare as inefficient. Also, I did not see the results of the LCRPGR in the rest of the paper, but only the LCPC

***Response 3***

Marked Line 236

Unmarked Line 222

Function modified to Land consumption per capita mean built up area divided by population

***Comment 4***

Line 204: although you listed the references that support the selection of independent variables, I suggest add some explanations, especially the theoretical underpinnings behind the potential associations.

***Response 4***

Marked line 112

Unmarked line 103

Section on theory added

Heading: Land cover change and consumption

***Comment 5***

Line 237: should be Table 3

***Response 5***

Marked line 312

Unmarked 300

Corrected to Table 3

***Comment 6***

Line 247, use cumulative “AS”, and the number should be 106580.8

***Response 6***

Corrected

***Comment 7***

Reformate Table 2 and Table 4 to fit the pages

***Response 7***

Tables made to fit

***Comment 8***

Line 278: the random forest modelling should be provided with more details. I am not an expert on random forest model and correct me if I am wrong. I understand that you were using the random forest to regress the correlations between land cover change and the influencing factors, and then using such correlations to predict the future change of land cover under “business as usual” scenario, however, more information should be provided, such as what is the target year of your prediction, have you set any thresholds of the influencing factors, such as the population growth (such as the world population projected by UN), climate change (maybe also from UN’s climate report?).

***Response 8***

Marked line 281-284

A basic explanation is provided

[creates a model based on known values from a training dataset and later uses it to forecast unknown values with the same associated explanatory factors. It generates a large number of decision trees, known as forest, each tree creates its own forecast, which is then utilised as part of a voting mechanism to determine final predictions] (Bühlmann, 2010).

Marked line 287-296

Unmarked line 274-278

Using the random forest regression, the future land consumption modelling generated 100 classification trees, 1 leaf size, 3 depth range of 4083 to 4404, and mean tree depth of 4269. The model also had 3 randomly sampled variables, with about 30% of the dataset excluded for validation. After training the data with the independent variables, prediction for 30 years future land cover for Greater Accra Region.

***Comment 9***

Line 283: the interpretation of the Fig 5, while I can tell from the Figure that population has the highest importance, but the text said poverty index has the highest level of importance

***Response 9***

Marked Line 347

Unmarked line 333

Rewriting: The factors used for the model generated various levels of predictive importance, with population having the highest level of importance, predicting land cover change by 21.19% while the least important variables was temperature (0.0%) (Fig 6).

***Comment 10***

I have read paper using population projection to predict land use change, or verse vise, but why do you think a random forest that includes all factors in the model is better?

***Response 10***

The factors which influence land cover change are theoretically and empirically anthropogenic and natural. Most studies depend on anthropogenic by using population growth but there are other factors as the modelling indicated distance from water bodies, poverty and distance from sacred groves influence growth. These are micro level factors which needs to be accounted for.

***Comment 11***

Line 303: it should be “Leo-Breiman

***Response 11***

Correction made

***Comment 12***

Line 342: the authors pointed out the planning and protection policies intensified the land consumption, but how to include such influences in the predicting model?

***Response 12***

Modelling policies was difficult

Proxy variables were distance from road, distance from government housing

***Comment 13***

What is the unit of analysis, especially when calculating the land efficiency? grid cells?

***Response 13***

Hectare from

<https://archive.unescwa.org/sites/www.unescwa.org/files/u593/module_3_land_consumption_edited_23-03-2018.pdf>

The grid cell was 30*30 meters because of the Landsat image used for classification

***Comment 14***

Actually, you were using the land consumption per capita (LCPC) from UN Habitat (<https://unhabitat.org/sites/default/files/2021/08/indicator_11.3.1_training_module_land_use_efficiency.pdf>) to calculate the “land use efficiency”, rather than the LCRPGR you mentioned (whether the increase of urbanized land is compatible with the increase of population, line 184). In addition, I did not find the threshold of low, moderately and efficient from the reference you provided (line 188, <https://apo.org.au/sites/default/files/resource-files/2018-07/apo-nid182836.pdf>).

***Response 14***

Mode to LCPC

Correction made in methodology

Interpretation for the levels of land efficiency can be found here

<https://archive.unescwa.org/sites/www.unescwa.org/files/u593/module_3_land_consumption_edited_23-03-2018.pdf>

**REVIEWER 2**

***Comment 1***

In the abstract part, studied methods and studied years (1987, 2005 and 2017) were not presented.

***Response 1***

The study adopted maximum likelihood image classification techniques and “combinatorial or “to model land cover change for Greater Accra from 1987 to 2017. While the UN-Habitat land efficiency index was employed to model land consumption and efficient of land cover change. Leo-Breiman Forest based regression was used to model future land cover by using the 30 years land cover change as dependant variable a series of natural and anthropogenic as independent variables.

***Comment 2***

Many minor problems need to be revised, such as the “Li, Ye, Song, 2020;” in line 47, “Fig 1” in line 139, “(Yan, Peng & Wu, 2020).” in line 111, “(Ahmad, Pandey, & Kumar, 2019).” In line 53, “Guo and Xiong, (2014)” in 114, “Liu et al,” in line 120, “Grekousis and Mountrakis, (2015)” in line 327, in line 349, “Fig 1: Map …” in line 147 and in all the figure captions.

***Response 2***

Corrected

***Comment 3***

Please unify the tense you used. Such as “Stewart and Haaga (2018) assert that increasing” in line 56-57, and all the primary tenses when you cited and described other reports.

***Response 3***

Well noted and corrected

***Comment 4***

This manuscript had two “Fig. 3” in line 207 and in line 252, respectively, pleased revise them as well as in the text.

***Response 4***

Tables and Figure numbers verified and corrected

***Comment 5***

In line 282-285, “…predicting land cover change by 13.91% while the least important variables were rainfall (9.53%) and slope (6.60%) …” why the data “13.91%, 9.53% and 6.60%” was disagreed with them in Fig. 5?

***Response 5***

The factors used for the Leo-Breiman forest based classification and regression model generated various levels of predictive importance, with population having the highest level of importance, predicting land cover change by 21.19% while the least important variables was temperature (0.0%) (Fig 6)

***Comment 6***

Line 251, delete the second “organic”

***Response 6***

No organic

***Comment 7***

Line 289, please revise “in at the experimental site”

***Response 7***

However, the explanatory variables had high validation shares within the range of 0.99 to 1 (Table 4).

**REVIEWER 3**

***Comment 1***

For which year land cover trends is simulated need to be precisely stated.

***Response 1***

Marked Line 201-202

Unmarked line 190-191

Data used for land cover mapping were the Landsat images of 1987, 2005 and 2017 for Greater Accra Region

***Comment 2***

Future trends of land consumption is evaluated using “business as usual scenario”. Why business as usual scenario was chosen for the analysis need to be properly justified. Given that it is unlikely to continue the existing growth rate for prolonged time. And, this model is susceptible for portraying an upper bound benchmark for urban growth.

***Response 2***

Marked line 291-296

Unmarked line 278-283

For the 30-year future land cover modelling, the study used the "business as usual" scenario, assuming that the forces influencing land cover change are semi-permanent and the result of all social and economic elements at play. Government has the greater influence to alter the trajectory of land cover change but with less influence on housing and land ownership since 1900’s such outcome is not expected (Gough & Yankson, 2000; Quarcoopome, 1992).

***Comment 3***

Few caption of table and tables were misplaced. Need to be corrected.

***Response 3***

Corrected
